# OD43 The Growing Clinical And Economical Burden Of Chronic Kidney Disease In Brazil: An IMPACT CKD Analysis

**DOI:** 10.1017/S0266462324001703

**Published:** 2025-01-07

**Authors:** Ricardo Moreira, Cassio Regis, Beatriz Grizolli, Cinthia Montenegro, Talita Gobbi, Naveen Rao, Stacey Priest, Stephen Brown, Hannah Guiang, Ana Flavia Moura, Ming-Hui Zhao, Steven Chadban

## Abstract

**Introduction:**

The growing burden of chronic kidney disease (CKD) in Brazil is increasingly evident, marked by its significant contributions to mortality rates and healthcare costs. Managing CKD, especially through renal replacement therapy (RRT), demands substantial resources. To enhance healthcare decision-making, a thorough examination of the relationship between the rising prevalence of CKD and its clinical and economic impacts is crucial.

**Methods:**

We developed a patient-level simulation model to project the natural history of CKD, defined as the IMPACT CKD. This model integrated factors such as acute kidney injury, cardiovascular events, and comorbidities, and aimed to assess CKD’s clinical, humanistic, and economic impact on the healthcare system. It forecasted the burden of CKD over the next decade (2023 to 2032). This projection is pivotal to derive the burden of CKD for health technology assessment (HTA) evaluations. Validation was conducted against Brazil’s demographic data and cross-validated with the Inside CKD model.

**Results:**

The IMPACT CKD forecast a rapid increase of CKD population in Brazil, outpacing the growth of the general population. Specifically, there is an expected 6.9 percent increase in stages 3 to 5 CKD, leading to a higher demand for dialysis (projected 370,000 cases in 2032) and transplants (projected 115,000 cases in 2032). A significant increase in cardiovascular CKD-related events (+100.6%) and mortality (+67.8%) is expected. In 2032, it is projected 15 million CKD patients will be in stages 1 to 2, and 12.7 million in stages 3 to 5. CKD-related healthcare costs will represent 25.7 percent of Brazil’s healthcare budget, and dialysis will reach USD2.7 billion in annual costs.

**Conclusions:**

IMPACT CKD predicts an increasing CKD prevalence and an alarming rise in stages 3 to 5 and RRT, including thousands of premature deaths, and a substantial economic burden on the Brazilian healthcare system. This data could be informative for healthcare decision-makers when choosing strategy to reduce the impact of CKD in Brazil.

